# Agmatinase promotes the lung adenocarcinoma tumorigenesis by activating the NO-MAPKs-PI3K/Akt pathway

**DOI:** 10.1038/s41419-019-2082-3

**Published:** 2019-11-07

**Authors:** Hui-er Zhu, Jia-yi Yin, De-xiong Chen, Sheng He, Hui Chen

**Affiliations:** 1 Department of Emergency Surgery, The Third Affiliated Hospital of Guangzhou Medical University, Guangzhou Medical University, Guangzhou, Guangdong 510150 PR China; 2 Department of Respiratory Medicine, The Third Affiliated Hospital of Guangzhou Medical University, Guangzhou Medical University, Guangzhou, Guangdong 510150 PR China; 3 Department of Pathology, The Third Affiliated Hospital of Guangzhou Medical University, Guangzhou Medical University, Guangzhou, Guangdong 510150 PR China

**Keywords:** Extracellular signalling molecules, Oncogenesis

## Abstract

Lung adenocarcinoma (LUAD) is one of the leading causes of cancer-related death worldwide. There is an urgent need to uncover the pathogenic mechanism to develop new treatments. Agmatinase (AGMAT) expression and its association with clinicopathological characteristics were analyzed via GEO, Oncomine, and TCGA databases, and IHC staining in human LUAD specimens. An EdU cell proliferation kit, propidiumiodide staining, colony formation, cell migration, and invasion assays, and a xenograft tumor model were used to detect the biological function of AGMAT in LUAD. Furthermore, the expression level of nitric oxide (NO) was detected using a DAF-FMDA fluorescent probe or nitrite assay kit, and further validated with Carboxy-PTIO (a NO scavenger). The roles of three isoforms of nitric oxide synthases (nNOS, eNOS, and iNOS) were validated using L-NAME (eNOS inhibitor), SMT (iNOS inhibitor), and spermidine (nNOS inhibitor). AGMAT expression was up-regulated in LUAD tissues. LUAD patients with high AGMAT levels were associated with poorer prognoses. AGMAT promoted LUAD tumorigenesis in NO released by iNOS both in vitro and in vivo. Importantly, NO signaling up-regulated the expression of cyclin D1 via activating the MAPK and PI3K/Akt-dependent c-myc activity, ultimately promoting the malignant proliferation of tumor cells. On the whole, AGMAT promoted NO release via up-regulating the expression of iNOS. High levels of NO drove LUAD tumorigenesis via activating MAPK and PI3K/Akt cascades. AGMAT might be a potential diagnostic and therapeutic target for LUAD patients.

## Introduction

Lung cancer is the most commonly diagnosed cancer and the most frequent cause of cancer-related death worldwide as reported by the GLOBOCAN 2018 estimates of cancer incidence and mortality produced by the International Agency for Research on Cancer^1^. There are 2.1 million new lung cancer cases and 1.8 million deaths predicted for 2018, representing close to 1 in 5 (18.4%) deaths due to cancer^1^. Lung cancer is divided into small cell lung cancer and non-small cell lung cancer^2,3^. NSCLC represents ~85% of all cases, of which lung adenocarcinoma (LUAD) is the most diagnosed histological subtype of NSCLC, followed by squamous cell carcinoma^2,4^. Although the main oncogenic drivers (e.g., EGFR, KRAS, and ALK) have been reported in LUAD over the past 20 years^5^, there is an urgent need to identify new therapeutic targets due to tumor heterogeneity.

Agmatinase (AGMAT) can inactivate agmatine, which functions as an intermediary in polyamine biosynthesis and is synthesized following the decarboxylation of L-arginine by arginine decarboxylase (ADC)^6^. Although AGMAT significantly affects the polyamine biosynthetic pathway^7^, it was not until 1994 that it was demonstrated that agmatine and agmatinase could be synthesized by mammals^8^. Agmatine has been shown to have a wide range of biological effects, including the inhibition of cell proliferation^9,10^, stimulation of the glomerular filtration rate^11^, and the regulation of nitric oxide synthase^12,13^. Thus, there is considerable interest in understanding the physiological mechanisms by which agmatine levels are regulated. Although AGMAT plays an important role in amino acid metabolism, there are limited reports on its effect in disease, especially in tumors. AGMAT expression is diminished in diabetic patients with breast cancer and renal clear cell carcinoma patients^14,15^. Moreover, AGMAT can promote the progression of colorectal cancer via inducing chronic inflammation^16^. However, the biological function, clinicopathological characteristics, and regulatory mechanism of AGMAT in LUAD remain poorly understood.

Endothelial nitric oxide synthase (eNOS), neuronal nitric oxide synthase (nNOS), and inducible nitric oxide synthase (iNOS) have been described as the three types of NO synthase isoenzymes in mammals^17,18^. Nitric oxide (NO) is a highly reactive radical and plays an important role in many physiological phenomena^19^. In addition, since NO provides sustained vascular tone maintenance and functions as a neural transmitter, it has become the primary research target in these areas^20^. In tumor cells, NO can stimulate angiogenesis and the dilation of arteriolar vessels^21^, as well as increase vascular permeability^22^. Mitogen-activated protein kinases (MAPKs) are signaling components that play an important role in complex cellular programs, including proliferation, differentiation, development, transformation, and apoptosis^23,24^. In addition, the MAPK pathway and nitric oxide synthases are closely interrelated, and NO can directly exert its function on the MAPKs^25^.

In the present study, we identified AGMAT as a key effector of the MAPK pathway in the malignant phenotypes of LUAD cells by regulating iNOS to produce NO. AGMAT is up-regulated in LUAD and is significantly associated with a poor prognosis in LUAD patients. The upregulation of AGMAT promoted LUAD cell growth, colony formation, cell cycle progression, the secretion of NO, while silencing AGMAT inhibited these effects. AGMAT significantly promoted the phosphorylation of Erk1/2, P38, and Akt, and upregulated the expression of cyclinD1. In addition, AGMAT was found to be a key influencing effector for the MAPK pathway to regulate malignant phenotypes of LUAD. This study indicates the functional roles of AGMAT in the development and progression of LUAD.

## Methods

### GEO, Oncomine, and TCGA data analysis

Three gene expression datasets (GSE10072^26^, GSE21933^27^, and GSE32863^28^) were downloaded from the Gene Expression Omnibus (http://www.ncbi.nlm.nih.gov/geo/) database. Oncomine (https://www.oncomine.org) is a public database of microarray profiles and next-generation sequencing. The TCGA LUAD and normal lung data set were retrieved from the TCGA database (provisional) using cBioPortal (http://www.cbioportal.org). A total of 515 LUAD patients and 59 normal lung tissue expression data were available for the analysis of AGMAT. For the survival and chi-square analyses, LUAD patients with low levels of AGMAT gene expression (the lower 30%; *n* = 155) were compared with those with high levels of gene expression (the upper 30%; *n* = 155).

### Gene set enrichment analysis (GSEA)

The global mRNA expression profiles of a subset of TCGA LUAD were performed using GSEA 2.0.9 software (http://www.broadinstitute.org/gsea/). For GSEA, AGMAT expression was used as a numeric variable. The estimated statistical significance of the ES before the enrichment scores for each set were normalized, a false discovery rate was counted, and the metric for ranking genes in GSEA was set as ‘Pearson’^22^.

### Tissue samples and immunohistochemistry analysis

A tissue microarray chip containing 50 pairs of LUAD tissue samples matched to their adjacent NT lung tissue samples was purchased from Alenabio (Xi’an, China). Paraffin-embedded sections were deparaffinized, rehydrated, and blocked, as previously described^29,30^. After incubating the sections with an anti-human monoclonal AGMAT antibody (1:100; sc-166414, Santa Cruz, TX, USA), they were incubated with a biotin-labeled mouse anti-goat antibody. The tissue microarray was visualized with streptavidin-conjugated horseradish peroxidase and 3,3-diaminobenzidine (DAB). According to the number of stained cells (≤5% positive cells, 0; 6–30% positive cells, 1; 31–50% positive cells, 2; 51–80% positive cells, 3; and >80% positive cells, 4) and the staining intensity (no staining, 0; mild staining, 1; moderate staining, 2; and severe staining, 3), each sample was scored. The final score was the sum of the percentage of positive cells and the staining intensity.

### Cells and cell cultures

The human LUAD cell lines, A549 and NCI-H1975, were purchased from the Cell Bank of the Chinese Academy of Sciences (Shanghai, China) and cultured in RPMI-1640 medium containing 10% fetal bovine serum (FBS). Hela and 293T cells were purchased from ATCC and cultured in DMEM medium containing 10% fetal bovine serum (FBS). All the cells were maintained at 37 °C in a 5% CO_2_ incubator.

### Vectors, retroviral infection, and transfection

The AGMAT expression construct was generated by PCR-amplified full-length human AGMAT cDNA into the pcDNA3.1-puro plasmid. Two shRNA sequences, shRNA#1 (CCATATTGCAAGCGATGGCAA) and shRNAi#2: (CAAACCCATTTATATCAGCTT), and a non-specific scrambled shRNA sequence, were constructed into a pSuper-retro-puro vector. A549 and NCI-H1975 cells were further transduced with pSuper-retro-puro-AGMAT shRNA or pSuper-retro-puro-Control shRNA for five days, after which the cells were selected with 2 μg/mL puromycin for 2 weeks. 293T cells were transfected into three plasmids (pCDH-AGMAT, psPAX2, and pMD2.G) to produce AGMAT–expressing lentiviruses.A549 and NCI-H1975 cells at 80% confluence were infected with empty or AGMAT–expressing lentiviruses using 5 μg/ml polybrene.

### Immunofluorescence staining

Following transfection with the AGMAT plasmid for 24 h, HeLa cells were plated onto glass coverslips for 24 h. After fixation in 4% paraformaldehyde, permeabilizing with 0.1% Triton X-100, the cells were incubated with anti-AGMAT antibodies or anti-Flag and subsequently incubated with the corresponding Cy3 or Alexa Fluor 488 conjugated secondary IgG antibodies. DAPI was used to stain the cellular nuclei.

### Quantitative real-time PCR

Total RNA was extracted from treated A549 and NCI-H1975 cells using the Trizol total RNA isolation reagent (Invitrogen). The level of AGMAT RNA was detected using qRT-PCR according to the manufacturer’s protocol. The level of AGMAT and GAPDH expression were measured using the 2−ΔΔCt method. The following primer sequences were used: AGMAT, forward: CTTGTCGAAGTTTCACCACCGTA, reverse: CTTTGGGGAGAGCACATAGCATC; and GAPDH, forward: CCCACTCCTCCACCTTTGAC, reverse: TCTTCCTCTTGTGCTCTTGC.

### Western blotting

The treated cells and tissues were lysed using lysis buffer (1 mM EDTA, 1% SDS, 5 mM DTT, 10 mM PMSF, 50 mM Tris–HCl pH 8.0, and a protease inhibitor cocktail). The total cell lysates were separated by 8% or 10% SDS-PAGE and then electroblotted onto PVDF membranes. Western blotting was performed using AGMAT (1:1000; sc-166414, Santa Cruz, TX, USA), Flag (1:2000; 20543-1-AP, proteintech, IL, USA) phospho-Erk1/2 (1:4000; #8544, CST, Boston, USA), Erk1/2 (1:4000; #4695, CST, Boston, USA), phospho-P38 (1:2000; #4511, CST, Boston, USA), P38 (1:2000; #8690, CST, Boston, USA), phospho-Akt (1:2000; #4060, CST, Boston, USA), Akt (1:2000; #4691, CST, Boston, USA), c-myc (1:2000; #13987, CST, Boston, USA) CyclinD1 (1:2000; 60186-1-Ig, proteintech, IL, USA), iNOS(1:2000; #2982, CST, Boston, USA), eNOS (1:2000; # 5880, CST, Boston, USA), eNOS (1:2000; # 4231, CST, Boston, USA), and β-actin (1:4000, sc-81178, Santa Cruz, TX, USA).

### Cell proliferation assay

A density of 1 × 10^4^ treated cells (overexpressing or stably silencing AGMAT) was seeded into 24-well plates. The cell number was counted at 24, 48, 72, 96, and 120 h. In addition, the treated cells (overexpressing or stably silencing AGMAT) were performed using an EdU Kit (C10310-1, RiboBio) to detect the capacity for proliferation according to the manufacturer’s protocol.

### Colony formation assays

For the colony formation assays, 600 treated A549 and NCI-H1975 cells (overexpressing or stably silencing AGMAT) were seeded and cultured in RPMI DMEM medium containing 10% FBS for 8 or 10 days, respectively. These clones were then fixed in methanol and stained with crystal violet solution.

### Cell cycle analysis

The treated cells (overexpressing or stably silencing AGMAT) were collected and fixed in 70% ethanol overnight at 4 °C. After washing three times with ice-cold PBS, the cells were resuspended in 500 μL PBS containing 50 μg/mL propidiumiodide (PI) for 30 min in the dark at room temperature. The cell cycle distribution was detected by flow cytometric analysis. Data were analyzed using ModFit software.

### Migration and invasion assays

Migration and invasion assays were performed using transwell chambers. For migration assay, 1 × 10^5^ LUAD cells stably overexpressing or silencing AGMAT were resuspended in RPMI 1640 medium in the upper transwell chambers. For invasion assay, 2 × 10^5^ LUAD cells stably overexpressing or silencing AGMAT were resuspended in RPMI 1640 medium with 0.1% FBS in the upper transwell chambers coated with Matrigel. The bottom chambers were placed in RPMI 1640 medium containing 10% FBS. The chambers were stained with 0.5% crystal violet. Migrated and invasive cells were detected with a microscope.

### Cell apoptosis assay

The cell apoptosis was measured using the Annexin V-PE/7-AAD apoptosis kit (MultiSciences, AP104). Briefly, 2 × 10^6^ NCI-H1975 and A549 cells overexpressing AGMAT or stably silencing AGMAT were washed two times with ice-cold PBS, incubated with 5 μL Annexin V-FITC and 10 μL 7-AAD for 5 min in the dark, and were detected by flow cytometry.

### Detection of NO production

Intracellular NO production was detected by DAF-FMDA (S0019, Beyotime, China). Following treatment, the cells were washed three times with ice-cold PBS and incubated with DAF-FMDA (5 mM) for 30 min at room temperature in the dark. The DAF-FM fluorescence intensity was detected using fluorescence microscopy.

Extracellular NO production was detected using an nitrite assay kit according to the manufacturer’s protocol (S0023, Beyotime, China). After treatment, 50 μL of the supernatants from each sample were collected and mixed with flavin adenine dinucleotide (FAD), nicotinamide adenine dinucleotide phosphate (NADPH), and nitrate reductase at 37 °C for 30 min. Then lactate dehydrogenase (LDH) and LDH buffer was added to each of the samples. After a 30 min incubation, 50 μL of Griess Reagent I and 50 μL of Griess Reagent II were added in the dark for 15 min, and the absorbance of each sample was detected using a microplate spectrophotometer at 540 nm. The level of NO in each sample was calculated according to the standard curve.

### In vivo tumor growth assay

A total of 6 × 10^6^ NCI-H1975/control shRNA or NCI-H1975/AGMAT shRNA#1 cells and a total of 4 × 10^6^ A549/control shRNA or A549/AGMAT shRNA#1 cells were subcutaneously injected into the right flanks of each BALB/c nu mouse (*n* = 6). After 21 days, all mice were killed, the tumors were stripped and weighed, and then subjected to immunohistochemical analysis and Western blotting.

### Statistical analysis

Data were presented as the mean ± standard deviation (SD) and was performed using GraphPad Prism 5. The survival curve was described by the Kaplan–Meier plot and was calculated using the log-rank test. An independent Student’s *t-*test (two-tailed) was applied to determine the statistical differences between the two groups. *p < 0.05; **p < 0.01; and ***p < 0.001 were considered statistically significant.

## Results

### AGMAT overexpression in LUAD correlates with clinicopathological features and prognosis

**Fig. 1:**
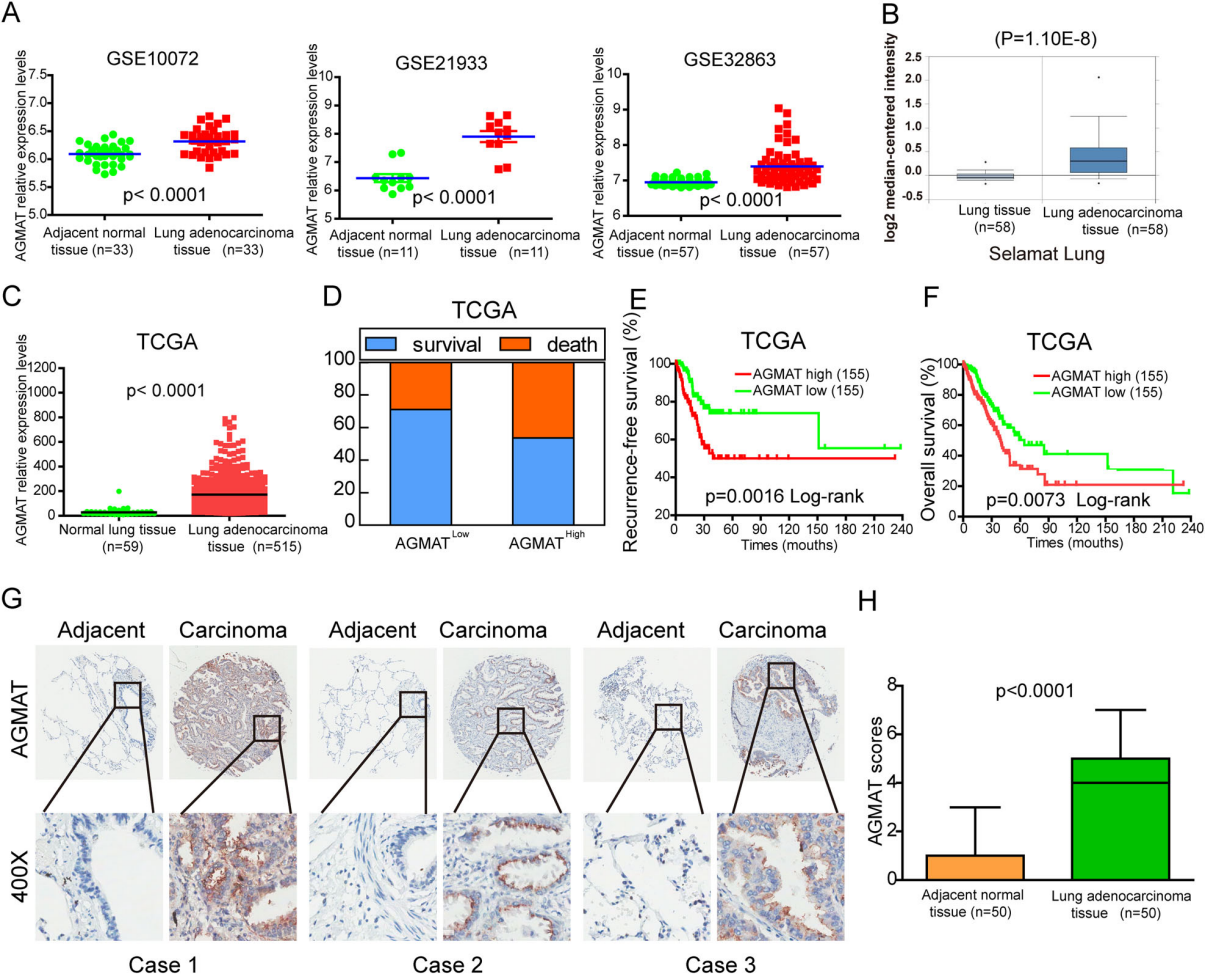
Upregulation of AGMAT is correlated with poor prognoses in LUAD. **a** Analysis of AGMAT mRNA expression in paired LUAD tissues from the three LUAD mRNA expression profiling datasets (GSE10072, GSE21933, and GSE32863). **b** Analysis of AGMAT mRNA expression in LUAD and normal lung tissues from the Selamat lung dataset based on the Oncomine database. **c** Analysis of the TCGA database for the levels of AGMAT mRNA expression in normal lung tissues and LUAD tissues. **d**–**f** Compared with the LUAD patients with a low level of AGMAT expression (the lower 30%), the patients with high AGMAT mRNA expression had higher death rates, shorter DFS, and OS from the TCGA LUAD specimen cohorts. **g** Representative IHC images of AGMAT expression in LUAD tissues and the corresponding adjacent normal lung tissues. **h** Differences in the AGMAT expression scores between LUAD tissues (*n* = 50) and adjacent normal lung tissues (*n* = 50) are shown as a box plot

Three publicly accessible microarray datasets of LUAD patients were analyzed for the level of AGMAT mRNA expression between LUAD tissues and their corresponding normal tissues. The analysis showed that the expression of AGMAT was significantly up-regulated in LUAD compared with the corresponding adjacent normal tissues (Fig. 1a). The Oncomine database was used to further analyze the level of AGMAT expression in other LUAD datasets, and the analysis in this database was surprisingly consistent with that of the GEO database (Fig. 1b, Fig. S1). In the TCGA database, the level of AGMAT gene expression in LUAD tissue was significantly higher than that in the normal lung tissue (Fig. 1c). To elucidate the relevance of AGMAT overexpression on the clinicopathological features of LUAD patients, we performed a Chi-square test. Our findings indicated that the high level of AGMAT mRNA expression was positively correlated with pT status (*P* = 0.004), pN status (*p* = 0.002), and clinical stage (*P* = 0.0003) (Table 1). To investigate the correlation of the level of AGMAT mRNA with the prognoses of LUAD patients, Kaplan–Meier survival analyses were performed. The results showed that patients with AGMAT overexpression trended to have higher death rates, shorter recurrence-free survival, and shorter overall survival (OS) (Fig. 1d, f). Furthermore, an extensive tissue microarray analysis of 50 pairs of matched LUAD and corresponding NT tissue samples was performed using an immunohistochemistry (IHC). We found that the level of AGMAT protein was significantly (*P* < 0.0001) up-regulated in LUAD compared with those in the corresponding adjacent normal tissues (Fig. 1g, h). These observations show that the level of AGMAT RNA and protein are significantly upregulated in LUAD, suggesting that AGMAT may serve as a prognostic molecular marker and a promoter of tumorigenesis.

**Table 1:** Comparison of clinical features between LUAD patients with low and high AGMAT levels in TCGA database

Clinical character	All cases	AGMAT		x^2^	*p* value
		High (*n* = 155) (%)	Low (*n* = 155) (%)		
Age (years)				0.015	0.901
≤60	95	48 (31.0)	47 (30.3)		
>60	203	101 (65.2)	102 (65.8)		
Gender^***^				17.71	<0.001
Male	163	100 (64.5)	63 (40.6)		
Female	147	55 (35.5)	92 (59.4)		
ALK translocation				0.004	0.948
No	128	65 (41.9)	63 (40.6)		
Yes	20	10 (6.5)	10 (6.5)		
pT status^**^				8.235	0.004
T1	90	33 (21.3)	57 (36.8)		
T2– T4	212	116 (74.8)	96 (61.9)		
pN status^**^				8.771	0.002
N0	198	87 (56.1)	111 (71.6)		
N1– N3	105	66 (42.6)	39 (25.2)		
pM status				0.355	0.551
M0	200	109 (70.3)	91 (58.7)		
M1	19	9 (5.8)	10 (6.5)		
Recurred/progressed				3.327	0.068
No	149	65 (41.9)	84 (54.2)		
Yes	105	58 (37.4)	47 (30.3)		
Clinical Stage^**^				8.989	0.003
Stage IA–IB	190	82 (52.9)	108 (69.7)		
Stage IIA–IV	115	70 (45.2)	45 (29.0)		

^#^American Joint Committee on Cancer classification (Version 7) (AJCC)

### AGMAT promotes the LUAD cell malignant phenotype

**Fig. 2:**
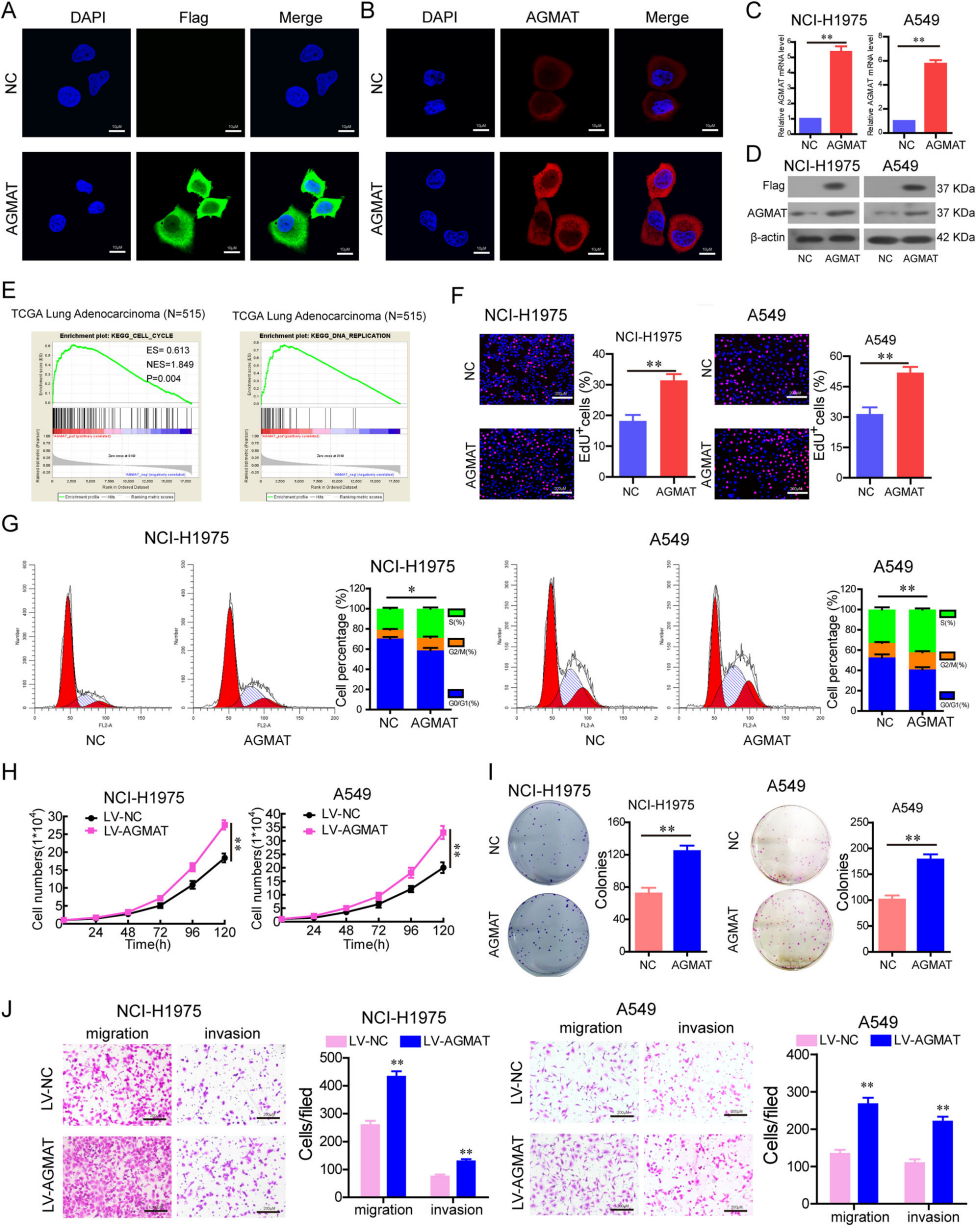
AGMAT promoted the malignant phenotypes of LUAD cells in vitro. **a**, **b** The indicated constructs were stably expressed in Hela cells, and Flag and AGMAT were immuno-stained using anti-Flag and AGMAT antibodies, respectively. **c**, **d** The overexpression of AGMAT was detected by RT-q-PCR and Western blot in NCI-H1975 and A549 cells. **e** GSEA plot indicating between DNA replication signatures and the level of AGMAT mRNA expression and between the cell cycle signatures and the level of AGMAT mRNA expression are significantly correlated in the TCGA adenocarcinoma specimen cohorts. **f**, **g** The effects of AGMAT transient overexpression on cell proliferation ability **f** and cell cycle **g** were detected in NCI-H1975 and A549 cells. **h**–**j** The effects of AGMAT stable overexpression on cell growth **h**, colony formation **i**, migration, and invasion **j** were detected in NCI-H1975 and A549 cells. Data are represented as mean ± SEM. *p < 0.05, **p < 0.01 or ***p < 0.001

To investigate the influence of the AGMAT on cancer progression, a construct was generated in which the Flag tag (six amino acids) was fused to the full-length AGMAT transcript. The indicated constructs were transfected into Hela cells for 24 h, and Flag and AGMAT immunostaining using anti-Flag and anti-AGMAT antibodies was performed. The results showed that Flag and AGMAT immunofluorescence staining were significantly increased in the AGMAT-Flag-transfected cells (Fig. 2a, b). Furthermore, the protein localization of AGMAT in Hela cells is consistent with previous IHC analysis. To indicate that the construct was overexpressed in NCI-H1975 and A549 cells, the level of AGMAT-Flag fusion protein expression was determined by RT-qPCR and Western blotting with anti-Flag and AGMAT antibodies (Fig. 2c, d).

To identify the possible functions of AGMAT, the global mRNA expression profiles of TCGA LUAD were performed using GSEA software. GSEA showed that the samples with high AGMAT expression were mainly enriched in the cell cycle gene sets and DNA replication gene sets, indicating that AGMAT may be closely related to cell proliferation (Fig. 2e). To investigate the role of AGMAT in LUAD, AGMAT was transiently transfected into NCI-H1975 and A549 cells. We found that the overexpression of AGMAT increased proliferation ability (Fig. 2f) and promoted cell cycle progression (Fig. 2g). In order to determine whether AGMAT involved in the effects on apoptosis of LUAD cells, Annexin V/7AAD kit was used to detect cell apoptosis. However, there was no significant difference of apoptosis rate between the overexpression of AGMAT group and NC group (Fig. S2). Next, we constructed LUAD cell lines stably overexpressing AGMAT by lentiviral vector (Fig. S3). We found that the stable overexpression of AGMAT promoted LUAD cells growth (Fig. 2h), colony formation (Fig. 2i), migration and invasion (Fig. 2j).

**Fig. 3:**
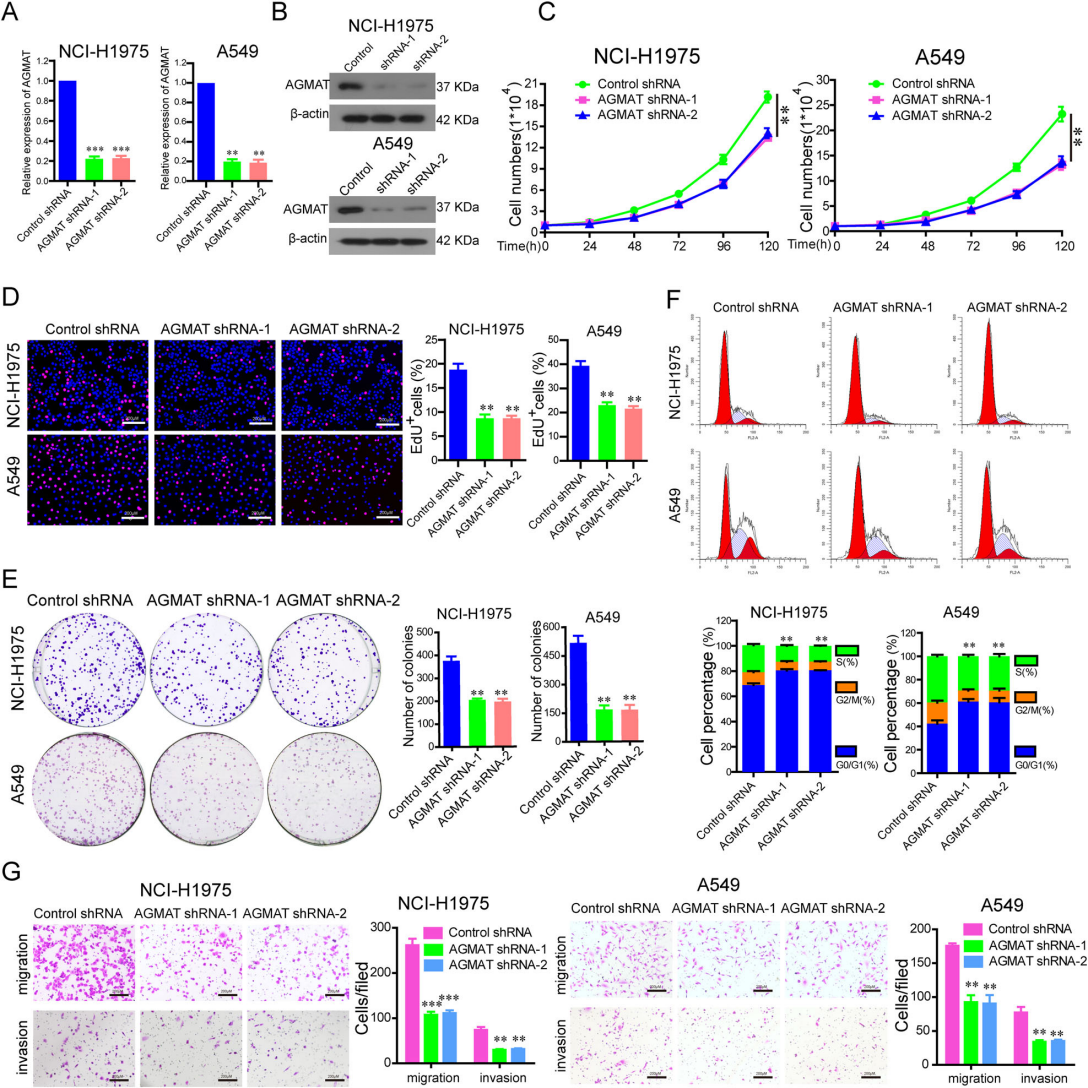
The stable silencing of AGMAT inhibits the malignant phenotypes of LUAD cells. **a**, **b** AGMAT expression was stably silenced in NCI-H1975 and A549 cells, which was detected by RT-q-PCR and Western blot. **c**–**g** The effects of stable AGMAT silencing on cell growth **c**, cell proliferation **d**, colony formation **e**, cell cycle progression **f**, migration, and invasion **g** were detected in NCI-H1975 and A549 cells. Data are represented as mean ± SEM. *p < 0.05, **p < 0.01 or ***p < 0.001

To further verify the role of AGMAT on the progression of LUAD, we constructed cell lines stably silencing AGMAT using shRNA. AGMAT mRNA and protein levels were significantly decreased when detected by qRT-PCR and Western blot (Fig. 3a, b). We observed that contrary to AGMAT overexpression, the stable silencing of AGMAT inhibited cell proliferation (Fig. 3c, d), colony formation (Fig. 3e), cell cycle progression (Fig. 3f), and migration and invasion (Fig. 3g). In addition, stably silencing AGMAT has no effect on apoptosis of NCI-H1975 and A549 cells compared to control group (Fig. S4).

### AGMAT significantly activates the MAPK and PI3K/Akt pathways through inducing NO production

**Fig. 4:**
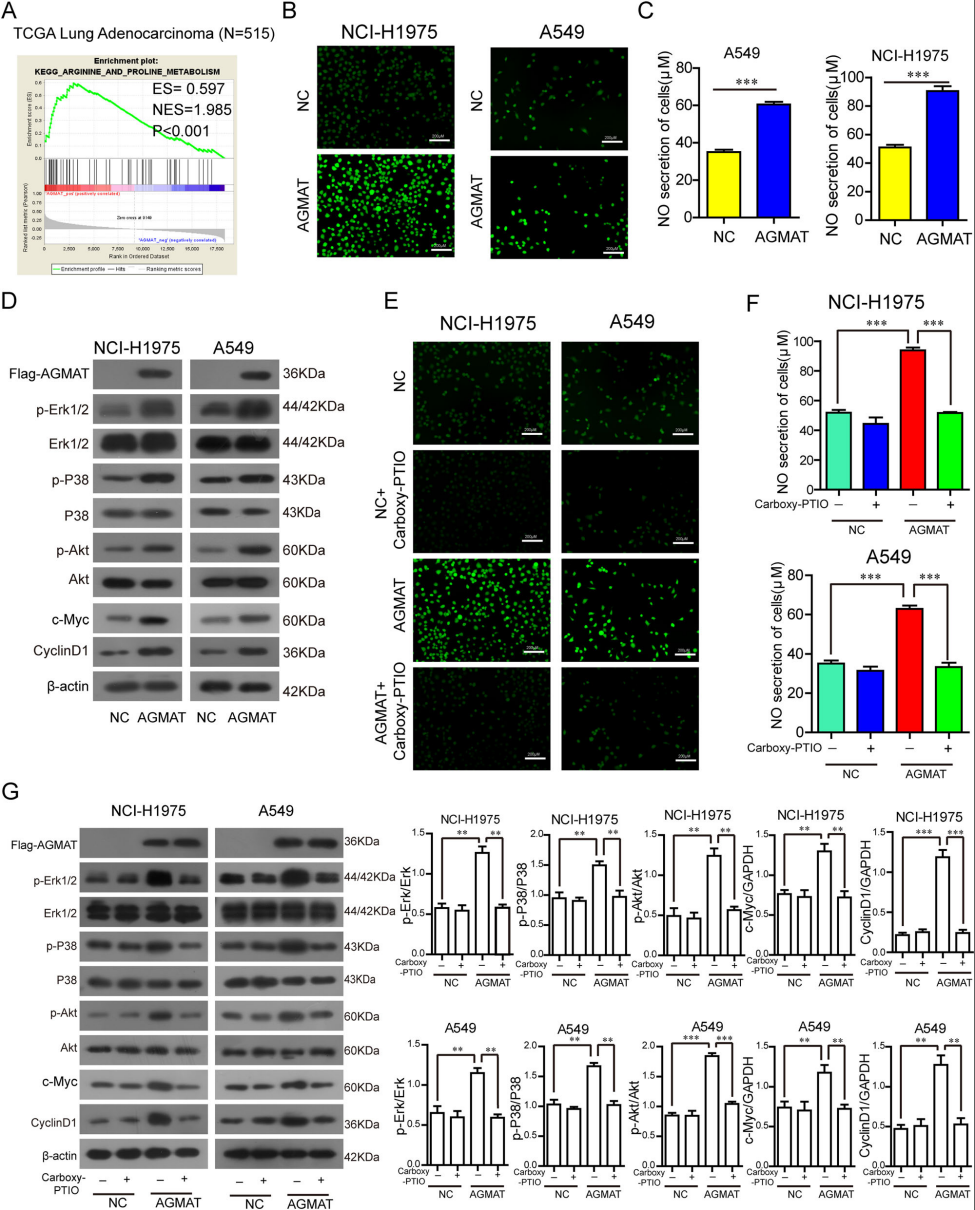
AGMAT activates the MAPK and PI3K/Akt pathway through inducing NO production. **a** GSEA plot indicating differences between the arginine and proline metabolism signatures and the level of AGMAT mRNA expression is significantly correlated in the TCGA adenocarcinoma specimen cohorts. **b**, **c** Following AGMAT overexpression for 36 h, the level of intracellular and extracellular NO was determined using DAF-FM DA staining and a nitrite assay kit, respectively. **d** Following AGMAT overexpression for 36 h, the level of phosphorylated Erk1/2, P38, and Akt, as well as the total level of Erk1/2, P38, Akt, AGMAT, c-myc, and cyclinD1 were determined by Western blot. **d**–**f** Following AGMAT overexpression for 36 h, the cells were treated with the NO scavenger, Carboxy-PTIO (100 μM) for 1 h. The level of intracellular **d** and extracellular **e** NO were determined. **f** The level of phosphorylated Erk1/2, P38, and Akt, and the total level of Erk1/2, P38, Akt, AGMAT, c-myc, and cyclinD1 were determined. Data are represented as mean ± SEM. *p < 0.05, **p < 0.01 or ***p < 0.001

To investigate how AGMAT promotes the malignant cell phenotype, GSEA was performed. The level of AGMAT mRNA expression and arginine metabolism gene signatures are closely related through GSEA (Fig. 4a). In addition, it was reported that AGMAT is involved in arginine metabolism^31^. NO is an important product in the process of arginine metabolism. Therefore, we detected whether changes in AGMAT expression had an effect on NO production. Intracellular NO production was detected by DAF-FMDA using a fluorescence microscope. The results showed that AGMAT overexpression in NCI-H1975 and A549 cells caused a substantial increase in the level of intracellular NO with enhanced fluorescence, whereas the cells in the control groups displayed low-density fluorescence (Fig. 4b). In addition, there was a higher level of NO secretion in the supernatants from NCI-H1975 and A549 cells overexpressing AGMAT, compared with the control group (Fig. 4c). NO is closely related to the MAPK and PI3K/Akt pathways. Here, we found that the up-regulation of AGMAT can stimulate the phosphorylation of Erk1/2, P38 and Akt, and increase the expression of c-myc and cyclinD1 in NCI-H1975 and A549 cells (Fig. 4d). The inhibitor of Erk1/2 kinases (U0126), P38 kinases (SB203580), Akt kinases (PF-04691502) and c-myc (10058-F4) were used to investigate the role of MAPK and PI3K/Akt pathways in regulating c-Myc activity and cyclin D expression. Compared to the vehicle-treated cells, treatment with U0126, SB203580, and PF-04691502 significantly reduced the phosphorylation of ERK1/2, P38 and Akt, respectively, also significantly reduced the expression of c-myc and cyclin D1 (Fig. S5A). In addition, compared to the vehicle-treated cells, treatment with 10058-F4 significantly reduced the expression of cyclin D1, and abolished AGMAT-induced up-regulation of cyclin D1 (Fig. S5B). In contrast, AGMAT silencing significantly reduced the level of intracellular and extracellular NO production (Fig. S6A, B), decreased the phosphorylation of Erk1/2, P38 and Akt, and the expression of c-myc and cyclinD1 (Fig. S6C). Carboxy-PTIO, a well-known NO scavenger, was used to further validate the role of NO in NCI-H1975 and A549 cells overexpressing AGMAT. The results showed that Carboxy-PTIO reversed the AGMAT overexpression induced by the phosphorylation of Erk1/2, P38 and Akt, and the upregulation of c-myc and cyclinD1 expression (Fig. 4e, g).

### AGMAT is a key effector of the MAPK and PI3K/Akt pathways by specifically regulating iNOS

**Fig. 5:**
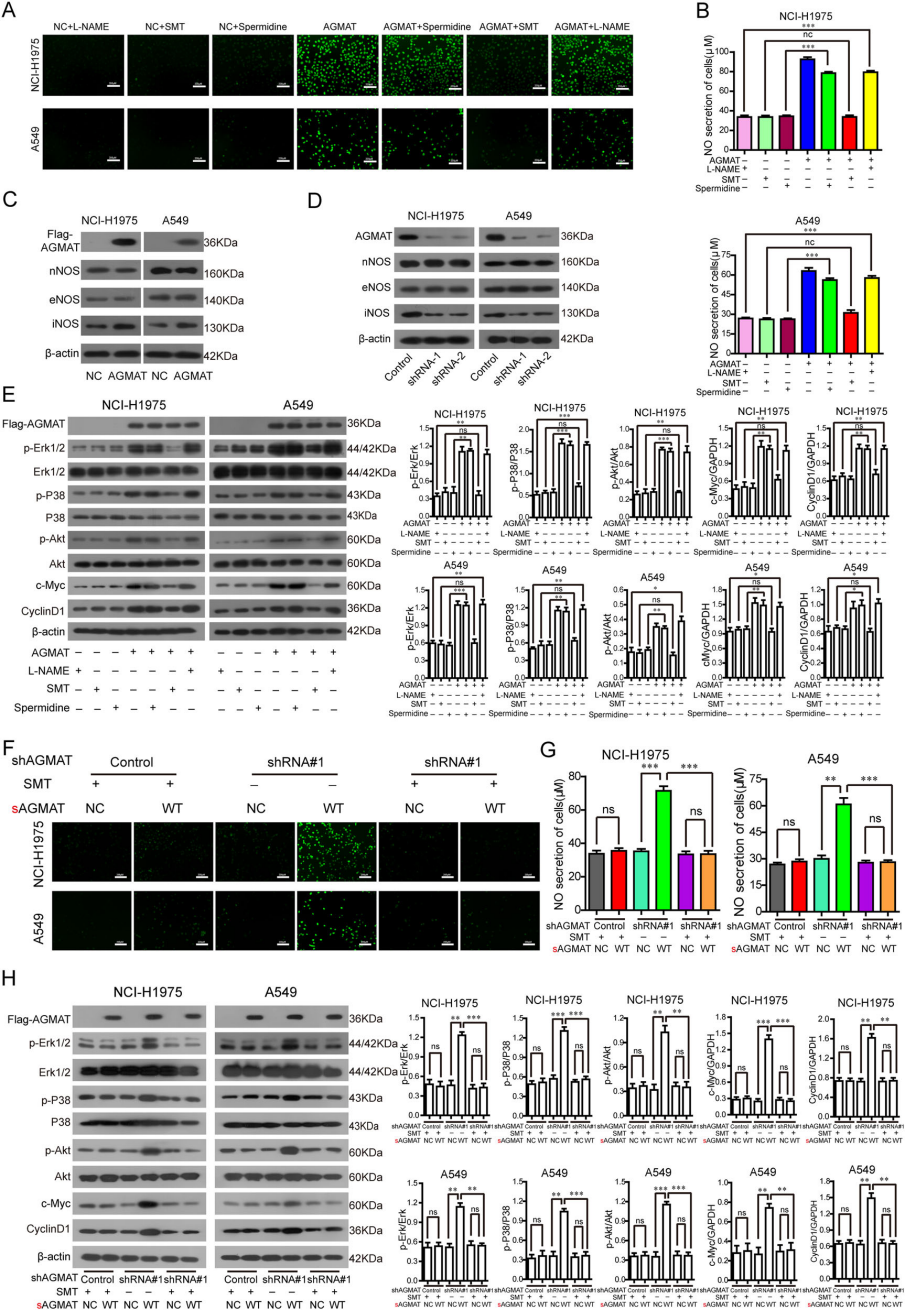
AGMAT activates the MAPK and PI3K/Akt pathways by specifically regulating iNOS expression. **a**, **b** After AGMAT overexpression for 24 h, the cells were treated with L-NAME (100 μM), or SMT (1 mM), or Spermidine (1 mM) for 24 h. The level of intracellular **a** and extracellular **b** NO was determined. **c**, **d** After AGMAT overexpression for 36 h or stably silencing, the level of nNOS, eNOS, and iNOS expression were detected by Western blot. **e** After AGMAT overexpression for 24 h, the cells were treated with L-NAME (100 μM), SMT (1 mM), or Spermidine (1 mM) for 24 h. The level of phosphorylated Erk1/2, P38, and Akt, and total level of Erk1/2, P38, Akt, AGMAT, c-myc, and cyclinD1 were determined. **f**–**h** The indicated synonymous mutant sAGMAT-Flag plasmids or its control plasmid was transfected into NCI-H1975 and A549 cells with stable silencing of AGMAT expression or into cells of the control group. After plasmid transfection for 24 h, the cells were treated SMT (1 mM), or for 24 h. The level of intracellular **f** and extracellular **g** NO was determined. **h** The level of phosphorylated Erk1/2, P38, and Akt, and total level of Erk1/2, P38, Akt, AGMAT, c-myc, and cyclinD1 were determined. Data are represented as mean ± SEM. *p < 0.05, **p < 0.01 or ***p < 0.001

To explore the mechanism of AGMAT promoting NO production, we applied three specific nitric oxide synthase inhibitors, L-NAME (eNOS Inhibitor), SMT (iNOS Inhibitor), and spermidine (nNOS Inhibitor). We found that SMT inhibited the secretion of both intracellular (Fig. 5a) and extracellular (Fig. 5b) NO caused by the overexpression of AGMAT. We also observed an increase in the expression of iNOS following AGMAT overexpression (Fig. 5c). The expression of iNOS was down-regulated in the in NCI-H1975 and A549 cells stably silencing AGMAT (Fig. 5d). In addition, the phosphorylation of Erk1/2, P38 and Akt, and the upregulation of c-myc and cyclinD1 expression caused by the upregulation of AGMAT were blocked by SMT in LUAD cells (Fig. 5e). To further prove that AGMAT specifically regulating iNOS, the synonymous mutant sAGMAT-Flag plasmids was constructed and applied to express the AGMAT protein in AGMAT-attenuated cells. We found that restoring the expression of AGMAT promoted intracellular (Fig. 5f) and extracellular (Fig. 5g) NO in AGMAT-attenuated cells. However, AGMAT expression could not enhance the secretion of both intracellular (Fig. 5f) and extracellular (Fig. 5g) NO in the presence of SMT in AGMAT-attenuated cells and corresponding control cells. SMT also inhibited the secretion of both intracellular (Fig. 5f) and extracellular (Fig. 5g) NO caused by the restoring the expression of AGMAT in AGMAT-attenuated cells. Moreover, the phosphorylation of Erk1/2, P38 and Akt, and the upregulation of c-myc and cyclinD1 expression caused by the restoring the expression of AGMAT were blocked by SMT in AGMAT-attenuated cells and corresponding control cells (Fig. 5h). Collectively, our results indicated that AGMAT is a key effector of the MAPK and PI3K/Akt pathways by specifically regulating iNOS.

### Stably silencing AGMAT reduced the growth of NCI-H1975 and A549 tumor xenografts in mice

**Fig. 6:**
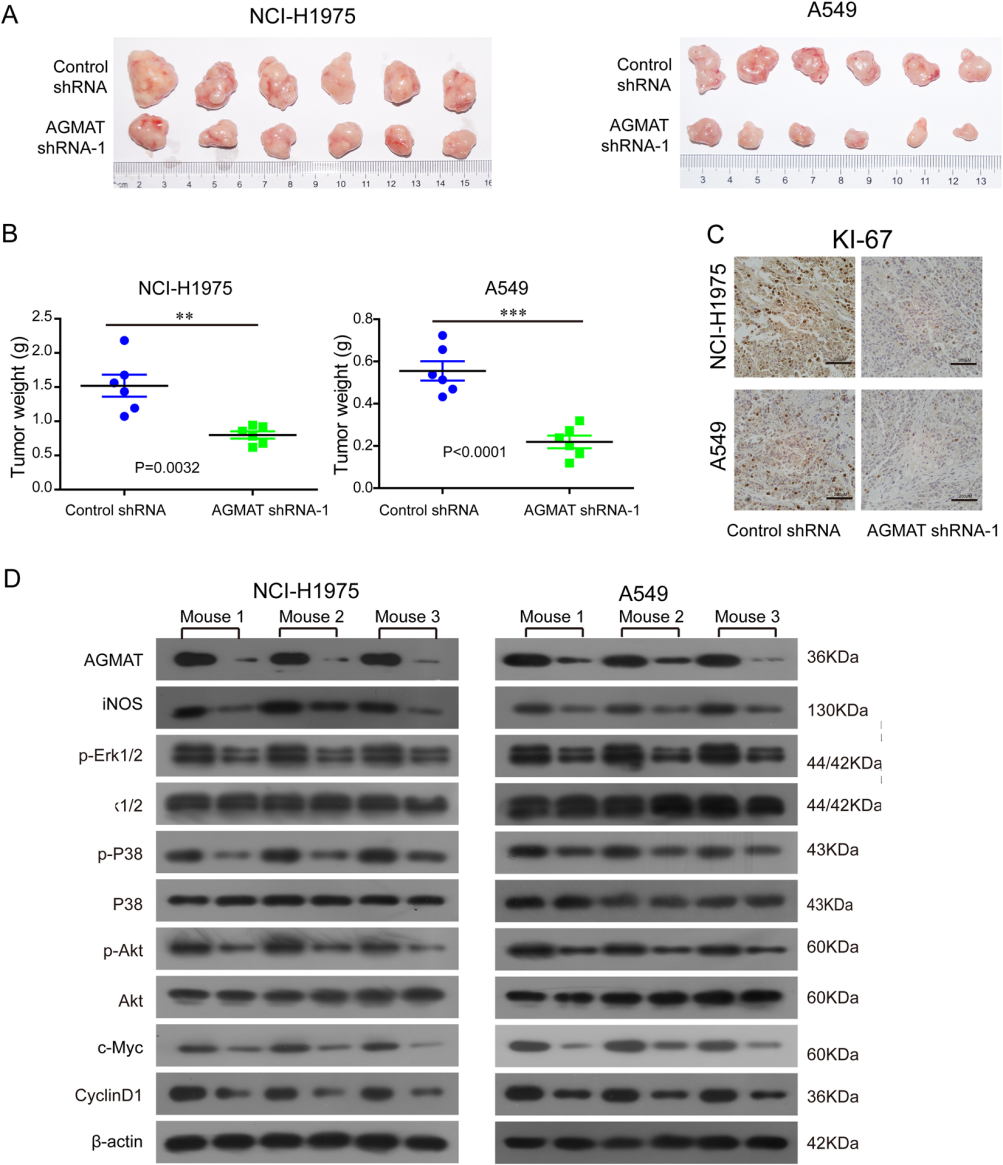
Silencing AGMAT inhibited the tumorigenicity of LUAD cells in vivo. **a** The in vivo growth of the NCI-H1975 and A549 cells lines stably silencing AGMAT were examined. Representative images of tumors in the indicated groups in nude mice were shown. **b** The weight of the xenograft tumors are presented (*n* = 6). **c** Representative images of Ki67 staining in tumors formed by the indicated cells are presented. **d** Tumors were lysed and the level of phosphorylated Erk1/2, P38, and Akt, and total level of Erk1/2, P38, Akt, AGMAT, iNOS, c-myc, and cyclinD1 were detected

BALB/c nude mice were subcutaneously injected with stably silenced AGMAT cells and control cells on the left and right flanks, respectively. The in vivo growth was clearly impaired in the NCI-H1975 and A549 xenografts composed of AGMAT-stably silenced cells compared with those composed of the control cells (Fig. 6a, b). Moreover, the Ki67 staining results were consistent with this phenomenon (Fig. 6c). The NCI-H1975 and A549 xenografts tumors were lysed. Compared with the control group, the level of Erk1/2, P38, and Akt phosphorylation, as well as the expression of iNOS, c-myc, and cyclinD1, was significantly decreased in the AGMAT-silenced group (Fig. 6d). Collectively, our results indicate that AGMAT promotes the LUAD cell malignant phenotypes in vivo, which was consistent with the in vitro results.

**Fig. 7:**
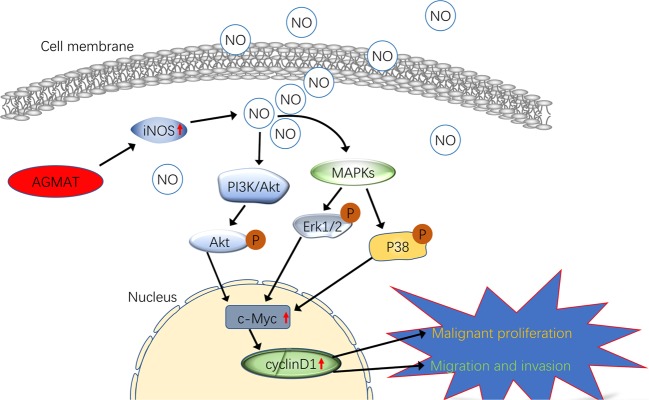
Schematic representation of the mechanism of agmatinase promotes the lung adenocarcinoma tumorigenesis

## Discussion

In the present study, we demonstrated that both the level of AGMAT mRNA and protein expression are up-regulated in LUAD tissues compared to the adjacent normal lung tissue by analyzing the GEO, Oncomine, and TCGA datasets, as well as a human LUAD tissue microarray. A Kaplan–Meier survival analysis revealed that patients overexpressing AGMAT were at a higher risk of LUAD-associated death than patients with low AGMAT expression, and they trended to have higher death rates and a shorter OS. The overexpression of AGMAT in human LUAD cell lines revealed that AGMAT promoted cellular proliferation, colony formation, migration, and invasion, while silencing AGMAT has the opposite effect. Furthermore, stably silencing AGMAT reduced the growth of NCI-H1975 and A549 tumor xenografts in mice. AGMAT helps LUAD cells to acquire malignant phenotype with increased proliferative capacity, motility, and invasiveness, leading to the in vivo growth of LUAD xenografts composed of AGAMT stably silencing cells was obviously impaired. In addition, we found that AGMAT could induce NO production via upregulating the expression of iNOS in H1975 and A549 cells. NO activates the MAPK and PI3K/Akt pathways, thereby up-regulating the expression of cyclinD1 protein, ultimately promoting the malignant proliferation of human LUAD cells. Previous studies have shown that AGMAT promotes the aggressive progression of colon cancer^16^. Our results obtained from LUAD are consistent with this finding, further supporting the tumor-promoting effect of AGMAT in various cancers. Arginine has important physiological and biochemical functions as the precursors of various compounds, including urea, NO, creatine, glutamate proline, and agmatine^32,33^. Arginine degradation occurs via multiple pathways under the effect of arginase, nitric-oxide synthase, and AGMAT^32,33^. These pathways produce NO, agmatine, glutamate, polyamines, and proline, with each having vital biological functions. In addition, abnormalities in these enzymes and enzymatic products often lead to some diseases, including vascular disease, pulmonary disease, infectious disease, and cancer. Several studies have found that agmatine has a significant anticancer effect. Agmatine was found to inhibit the proliferation of six colon cancer cell lines in a concentration-dependent manner, and the agmatine content was low in colon carcinoma tissue specimens^34^. Agmatine also inhibited the proliferation of tumor cells of colonic, hepatic, and neuronal origins^35^. Moreover, exogenous agmatine can also inhibit the proliferation of human leukemia HMC-1 and HL-60 cells^36^. In addition, agmatine is responsible for the conversion of agmatine to putrescine in vivo^37^. Agmatine not only prevented the LPS-induced elevation in mRNA expression of iNOS but also decreased the activity of iNOS induced by LPS, thereby inhibiting the production of nitrite^38,39^. In addition, it was also found in acute kidney injury models that agmatine decreased the levels of renal NO and iNOS^40^. Therefore, based on the combination of our results and the published literature, we speculate that AGMAT promotes iNOS expression and NO production by hydrolyzing agmatine, thereby promoting the development of lung adenocarcinoma. Whether AGMAT inhibits the anticancer effect of agmatine by hydrolyzing agmatine, thereby exhibiting a cancer-promoting effect, will be the focus of our future research.NO/iNOS are closely related to the MAPK and PI3K/Akt pathways. Hwanglyeonhaedok-tang, a traditional medicine used for the treatment of gastritis, was found to decrease iNOS expression to inhibit the activation of MAPK and PI3K/Akt pathways in gastric cancer cells^41^. Moreover, rifaximin treatment downregulated the release of NO, and resulted in a concentration-dependent decrease in the phosphorylation of Akt, mTOR, and p38MAPK^42^. We found that AGMAT promoted NO release via up-regulating iNOS expression to activate the MAPK and PI3K/Akt signaling cascades. In addition, NO generated by iNOS can protect tumor cells from apoptosis induced by chemotherapeutic drugs^43,44^. However, overexpression AGMAT or stably silencing AGMAT has no effect on apoptosis of NCI-H1975 and A549 cells. We speculate that AGMAT may have an effect on apoptosis of LUAD cells only when external adverse factors exist, such as chemotherapeutic drugs. This interesting phenomenon will be our next research work.

In summary, AGMAT up-regulation was correlated with poor prognoses in patients with LUAD. A novel role for AGMAT in LUAD tumorigenesis was elucidated, in which the upregulation of AGMAT promotes cell growth, cellular proliferation, colony formation, and cell cycle progression (Fig. 7). Moreover, AGMAT promotes NO release via upregulating the expression of iNOS in LUAD cells. Furthermore, AGMAT is a key effector of the MAPK and PI3K/Akt pathways through NO generated by iNOS (Fig. 7). Thus, AGMAT may serve as a novel prognostic factor and therapeutic target for LUAD.

## Supplementary information

Supplemental Figure captions

Supplementary Figure 1

Supplementary Figure 2

Supplementary Figure 3

Supplementary Figure 4

Supplementary Figure 5

Supplementary Figure 6

## References

[1] Bray F (2018). Global cancer statistics 2018: GLOBOCAN estimates of incidence and mortality worldwide for 36 cancers in 185 countries. CA: a Cancer J. Clin..

[2] Roca E (2017). Outcome of patients with lung adenocarcinoma with transformation to small-cell lung cancer following tyrosine kinase inhibitors treatment: a systematic review and pooled analysis. Cancer Treat. Rev..

[3] Xiao Y (2015). Epigenetic regulation of miR-129-2 and its effects on the proliferation and invasion in lung cancer cells. J. Cell. Mol. Med..

[4] Liang W (2015). Development and validation of a nomogram for predicting survival in patients with resected non-small-cell lung cancer. J. Clin. Oncol..

[5] Calvayrac Olivier, Pradines Anne, Pons Elvire, Mazières Julien, Guibert Nicolas (2017). Molecular biomarkers for lung adenocarcinoma. European Respiratory Journal.

[6] Reis DJ, Regunathan S (2000). Is agmatine a novel neurotransmitter in brain?. Trends Pharmacol. Sci..

[7] Laube G, Bernstein HG (2017). Agmatine: multifunctional arginine metabolite and magic bullet in clinical neuroscience?. Biochem. J..

[8] Li G (1994). Agmatine: an endogenous clonidine-displacing substance in the brain. Science.

[9] Peitsch MC (1996). ProMod and Swiss-Model: Internet-based tools for automated comparative protein modelling. Biochem. Soc. Trans..

[10] Igarashi K, Kashiwagi K (2000). Polyamines: mysterious modulators of cellular functions. Biochem. Biophys. Res. Commun..

[11] Sastre M, Regunathan S, Reis DJ (1997). Uptake of agmatine into rat brain synaptosomes: possible role of cation channels. J. Neurochem..

[12] Singh R, Pervin S, Karimi A, Cederbaum S, Chaudhuri G (2000). Arginase activity in human breast cancer cell lines: N(omega)-hydroxy-L-arginine selectively inhibits cell proliferation and induces apoptosis in MDA-MB-468 cells. Cancer Res..

[13] Wu G, Morris SM (1998). Arginine metabolism: nitric oxide and beyond. Biochem. J..

[14] Dallmann K (2004). Human agmatinase is diminished in the clear cell type of renal cell carcinoma. Int. J. Cancer.

[15] Celik VK (2017). Serum levels of polyamine synthesis enzymes increase in diabetic patients with breast cancer. Endocr. Connect..

[16] Snezhkina AV (2016). The dysregulation of polyamine metabolism in colorectal cancer is associated with over-expression of c-myc and C/EBPbeta rather than Enterotoxigenic Bacteroides fragilis. Infect. Oxid. Med. Cell. Longev..

[17] Garcia-Ortiz A, Serrador JM (2018). Nitric oxide signaling in T cell-mediated immunity. Trends Mol. Med..

[18] Zhao S (2016). Shengjing capsule improves erectile function through regulation of nitric oxide-induced relaxation in corpus cavernosum smooth muscle in a castrated rat model. Urology.

[19] Palmer RM, Ferrige AG, Moncada S (1987). Nitric oxide release accounts for the biological activity of endothelium-derived relaxing factor. Nature.

[20] Wang W, Lee Y, Lee CH (2015). Effects of nitric oxide on stem cell therapy. Biotechnol. Adv..

[21] Marrogi AJ (2000). Nitric oxide synthase, cyclooxygenase 2, and vascular endothelial growth factor in the angiogenesis of non-small cell lung carcinoma. Clin. Cancer Res..

[22] Joshi MS (2007). Receptor-mediated activation of nitric oxide synthesis by arginine in endothelial cells. Proc. Natl Acad. Sci. USA.

[23] Cargnello M, Roux PP (2011). Activation and function of the MAPKs and their substrates, the MAPK-activated protein kinases. Microbiol. Mol. Biol. Rev..

[24] Li A (2017). S100A6 promotes cell proliferation in human nasopharyngeal carcinoma via the p38/MAPK signaling pathway. Mol. Carcinogenesis.

[25] Lander HM, Jacovina AT, Davis RJ, Tauras JM (1996). Differential activation of mitogen-activated protein kinases by nitric oxide-related species. J. Biol. Chem..

[26] Landi MT (2008). Gene expression signature of cigarette smoking and its role in lung adenocarcinoma development and survival. PLoS ONE.

[27] Lo FY (2012). The database of chromosome imbalance regions and genes resided in lung cancer from Asian and Caucasian identified by array-comparative genomic hybridization. BMC Cancer.

[28] Selamat SA (2012). Genome-scale analysis of DNA methylation in lung adenocarcinoma and integration with mRNA expression. Genome Res..

[29] Zhu H, Chen H, Wang J, Zhou L, Liu S (2019). Collagen stiffness promoted non-muscle-invasive bladder cancer progression to muscle-invasive bladder cancer. OncoTargets Ther..

[30] Lin Q, Chen H, Zhang M, Xiong H, Jiang Q (2019). Knocking down FAM83B inhibits endometrial cancer cell proliferation and metastasis by silencing the PI3K/AKT/mTOR pathway. Biomed. Pharmacother..

[31] Wang X (2014). Arginine decarboxylase and agmatinase: an alternative pathway for de novo biosynthesis of polyamines for development of mammalian conceptuses. Biol. Reprod..

[32] Wu G (2009). Arginine metabolism and nutrition in growth, health and disease. Amino acids.

[33] Morris SM (2009). Recent advances in arginine metabolism: roles and regulation of the arginases. Br. J. Pharmacol..

[34] Molderings GJ (2004). Intestinal tumor and agmatine (decarboxylated arginine): low content in colon carcinoma tissue specimens and inhibitory effect on tumor cell proliferation in vitro. Cancer.

[35] Wolf C (2007). Molecular basis for the antiproliferative effect of agmatine in tumor cells of colonic, hepatic, and neuronal origin. Mol. Pharmacol..

[36] Haenisch B (2011). Effects of exogenous agmatine in human leukemia HMC-1 and HL-60 cells on proliferation, polyamine metabolism and cell cycle. Leuk. Res..

[37] Iyer RK, Kim HK, Tsoa RW, Grody WW, Cederbaum SD (2002). Cloning and characterization of human agmatinase. Mol. Genet. Metab..

[38] Ahn SK (2012). Protective effects of agmatine on lipopolysaccharide-injured microglia and inducible nitric oxide synthase activity. Life Sci..

[39] El-Awady MS, Nader MA, Sharawy MH (2017). The inhibition of inducible nitric oxide synthase and oxidative stress by agmatine attenuates vascular dysfunction in rat acute endotoxemic model. Environ. Toxicol. Pharmacol..

[40] Sharawy MH, Abdelrahman RS, El-Kashef DH (2018). Agmatine attenuates rhabdomyolysis-induced acute kidney injury in rats in a dose dependent manner. Life Sci..

[41] Park HS (2018). Gastroprotective effects of Hwanglyeonhaedok-tang against Helicobacter pylori-induced gastric cell injury. J. Ethnopharmacol..

[42] Esposito G (2016). Rifaximin, a non-absorbable antibiotic, inhibits the release of pro-angiogenic mediators in colon cancer cells through a pregnane X receptor-dependent pathway. Int. J. Oncol..

[43] Fahey JM, Stancill JS, Smith BC, Girotti AW (2018). Nitric oxide antagonism to glioblastoma photodynamic therapy and mitigation thereof by BET bromodomain inhibitor JQ1. J. Biol. Chem..

[44] Perrotta C (2018). Nitric oxide generated by tumor-associated macrophages is responsible for cancer resistance to cisplatin and correlated with syntaxin 4 and acid sphingomyelinase inhibition. Front. Immunol..

