# Supplementary material for: Agmatinase promotes the lung adenocarcinoma tumorigenesis by activating the NO-MAPKs-PI3K/Akt pathway
Source: Cell Death Dis. 2019 Nov 7;10(11):854. doi: 10.1038/s41419-019-2082-3 (PMC6838094; doi:10.1038/s41419-019-2082-3)
Supplement: Supplementary file 1 — Supplemental Figure captions [file 41419_2019_2082_MOESM1_ESM.docx]

**Supplemental Figure captions**

**Supplementary Figure 1.** Analysis of AGMAT mRNA expression in LUAD tissues and normal lung tissues from Hou lung, Su lung and Selamat lung data sets based on the Oncomine database

**Supplementary Figure 2.** The effect of AGMAT transient overexpression on NCI-H1975 and A549 cells apoptosis was detected by flow cytometry. Data are represented as mean ± SEM. *p<0.05, **p<0.01 or ***p<0.001.

**Supplementary Figure 3.** NCI-H1975 and A549 cell lines stably overexpressing AGMAT were constructed by lentiviral vector. (A) The expression of GFP was observed by fluorescence microscopy. (B) The AGMAT levels were detected by western blotting in NCI-H1975 and A549 cells stably overexpressing AGMAT.

**Supplementary Figure 4.** The effect of stable silencing AGMAT on NCI-H1975 and A549 cells apoptosis was detected by flow cytometry. Data are represented as mean ± SEM. *p<0.05, **p<0.01 or ***p<0.001.

**Supplementary Figure 5.** Effect of AGMAT on cyclinD1 expression in NCI-H1975 cells was detected in presence of inhibitors of Erk1/2, P38, Akt, and c-Myc**.** (A) After AGMAT overexpression for 24 h, the cells were treated with U0126 (10 μM), SB203580 (10 μM), or PF-04691502 (20 nM) for 24 h. The level of phosphorylated Erk1/2, P38, and Akt, and total level of Erk1/2, P38, Akt, AGMAT, c-Myc, and cyclinD1 were determined. (B) After AGMAT overexpression for 24 h, the cells were treated with 10058-F4 (200 μM) for 24 h, total level of AGMAT, c-Myc, and cyclinD1 were detected. Data are represented as mean ± SEM. *p<0.05, **p<0.01 or ***p<0.001.

**Supplementary Figure 6.** Silencing AGMAT reduced NO production to inhibit the MAPK and PI3K/Akt pathways. (A and B) The level of intracellular (A) and extracellular (B) NO in NCI-H1975 and A549 cells stably silencing AGMAT were determined using DAF-FM DA staining and a nitrite assay kit, respectively. (C) The level of phosphorylated Erk1/2, P38, and Akt, and total level of Erk1/2, P38, Akt, AGMAT, c-Myc, and cyclinD1 in NCI-H1975 and A549 cells stably silencing AGMAT were detected by Western blot.
