# Supplementary figures and images for: Agmatinase promotes the lung adenocarcinoma tumorigenesis by activating the NO-MAPKs-PI3K/Akt pathway
Source: Cell Death Dis. 2019 Nov 7;10(11):854. doi: 10.1038/s41419-019-2082-3 (PMC6838094; doi:10.1038/s41419-019-2082-3)

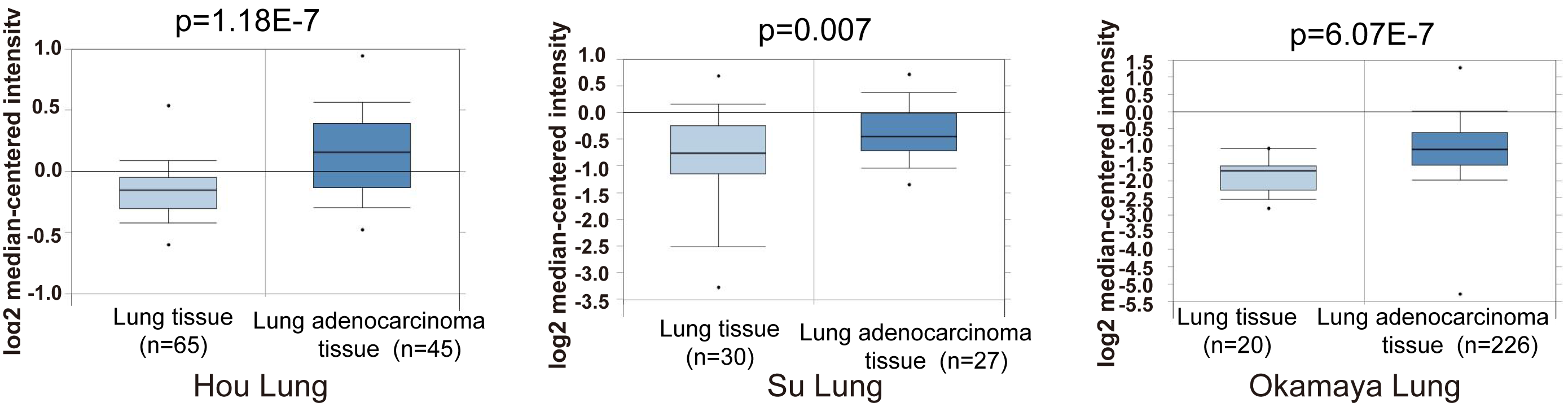

Supplement: Supplementary file 2 — Supplementary Figure 1 [file 41419_2019_2082_MOESM2_ESM.tif]

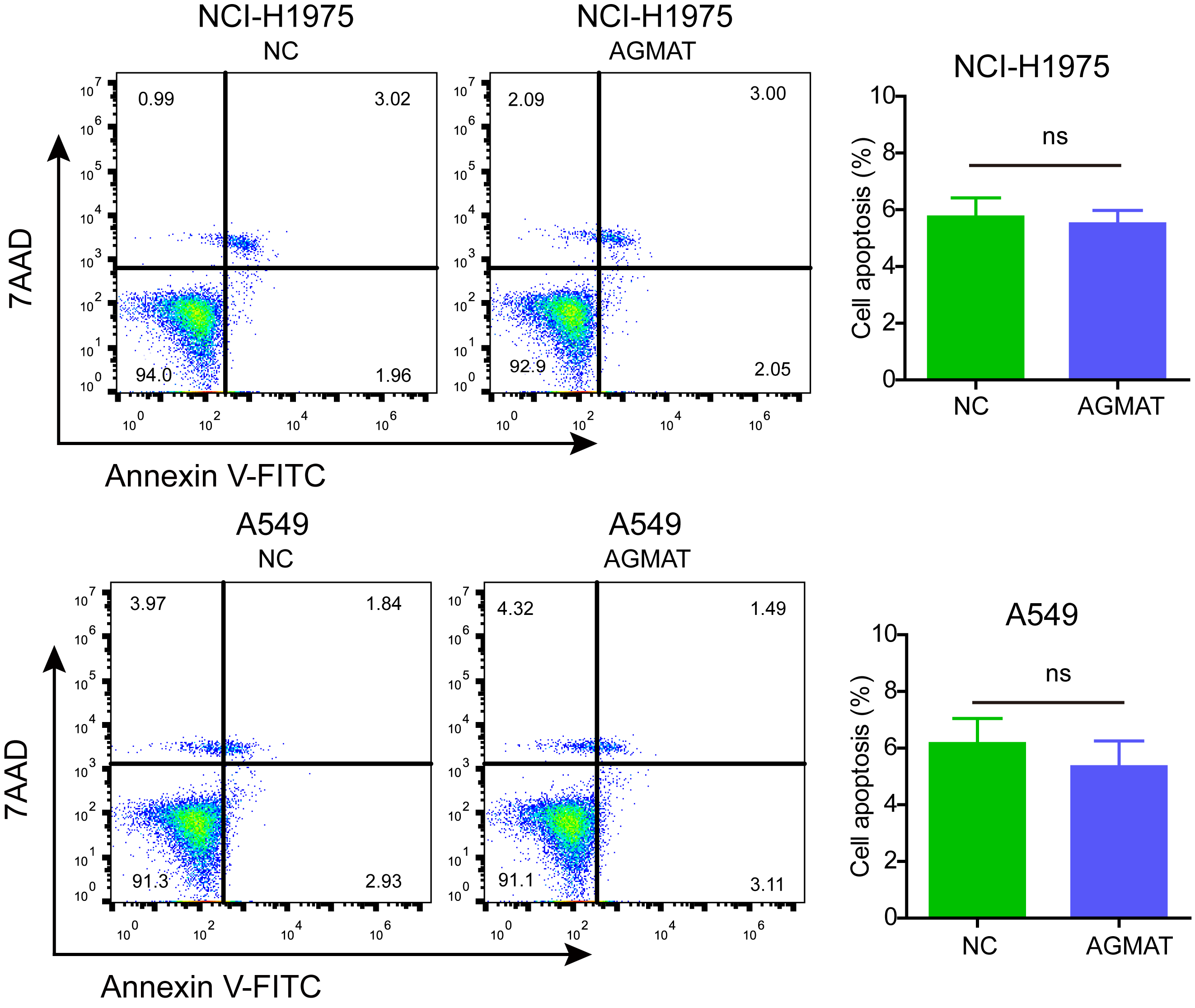

Supplement: Supplementary file 3 — Supplementary Figure 2 [file 41419_2019_2082_MOESM3_ESM.tif]

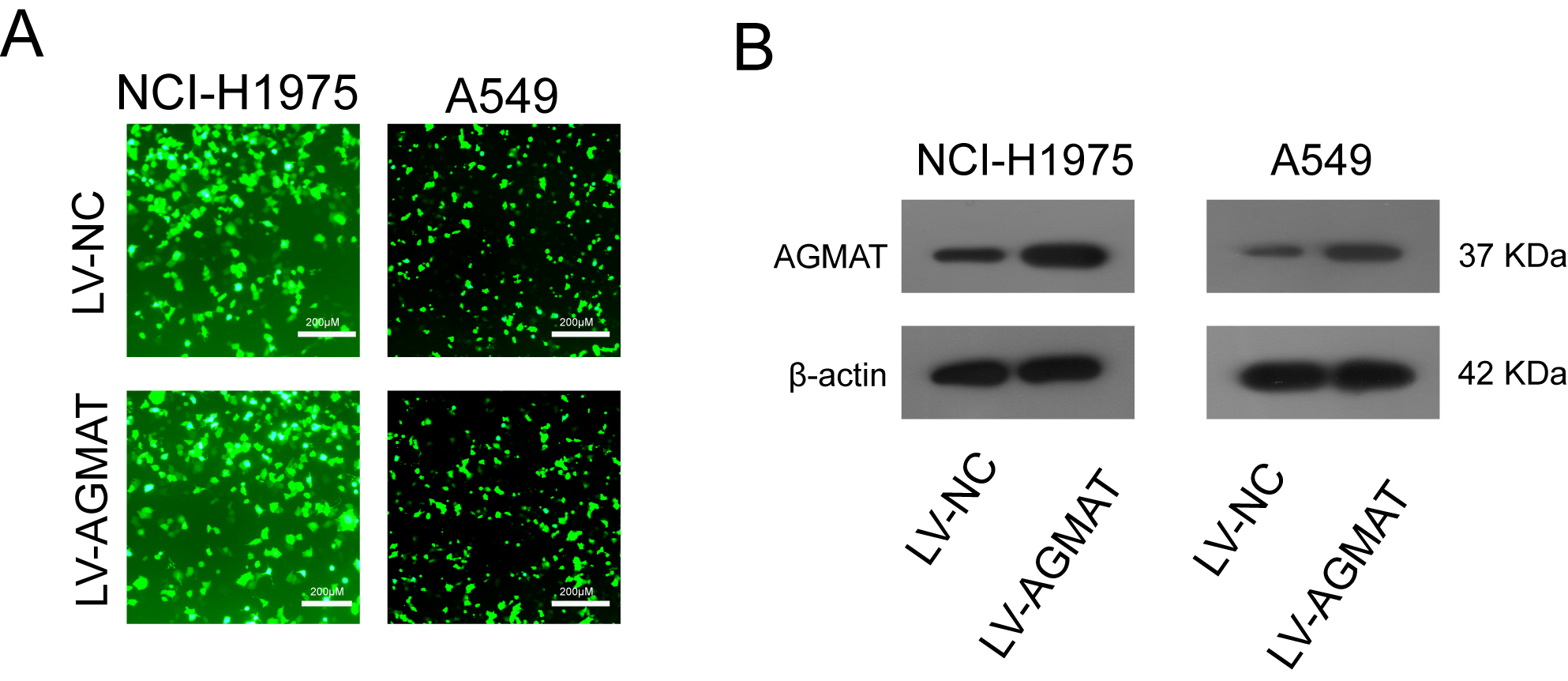

Supplement: Supplementary file 4 — Supplementary Figure 3 [file 41419_2019_2082_MOESM4_ESM.tif]

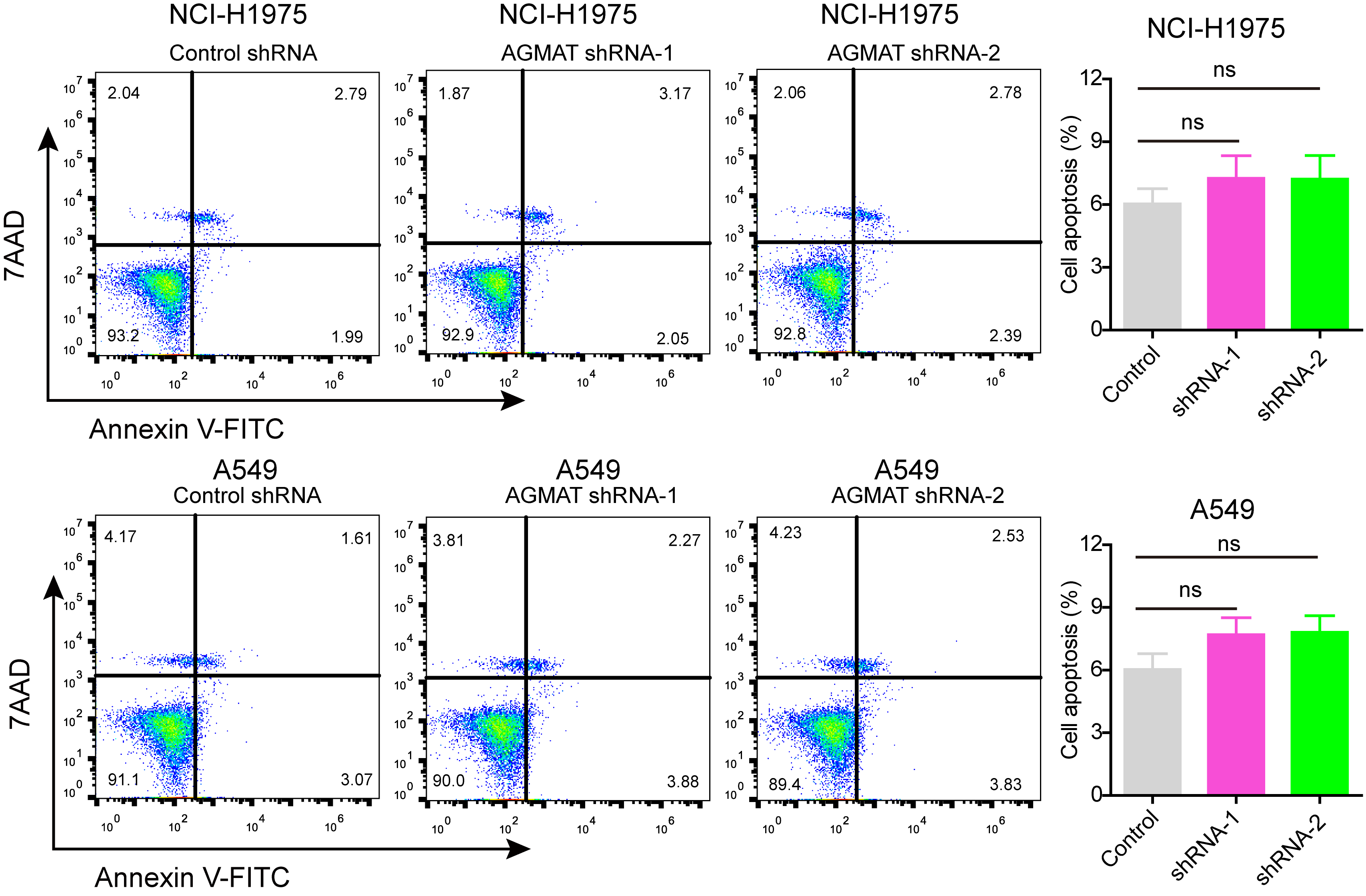

Supplement: Supplementary file 5 — Supplementary Figure 4 [file 41419_2019_2082_MOESM5_ESM.tif]

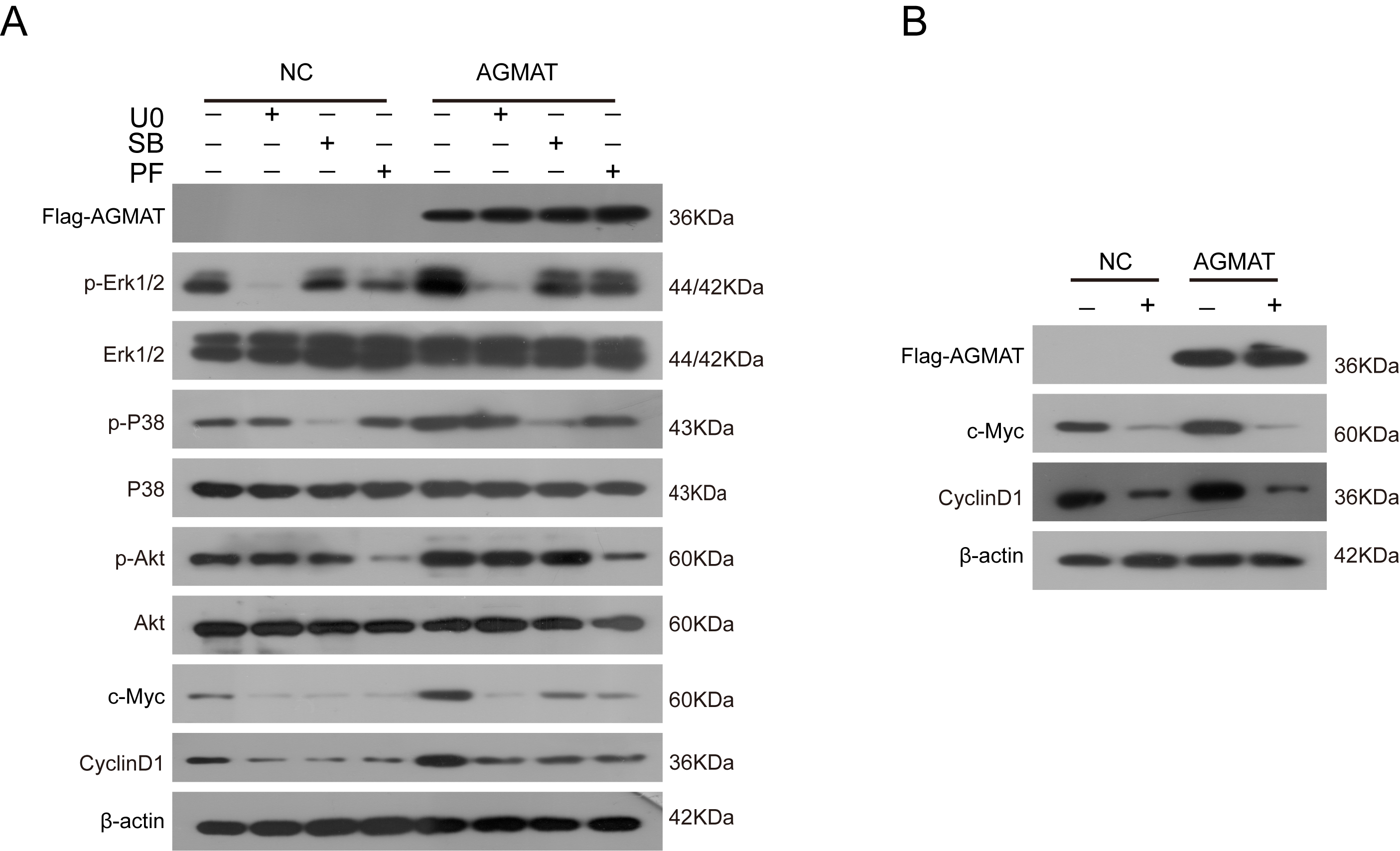

Supplement: Supplementary file 6 — Supplementary Figure 5 [file 41419_2019_2082_MOESM6_ESM.tif]

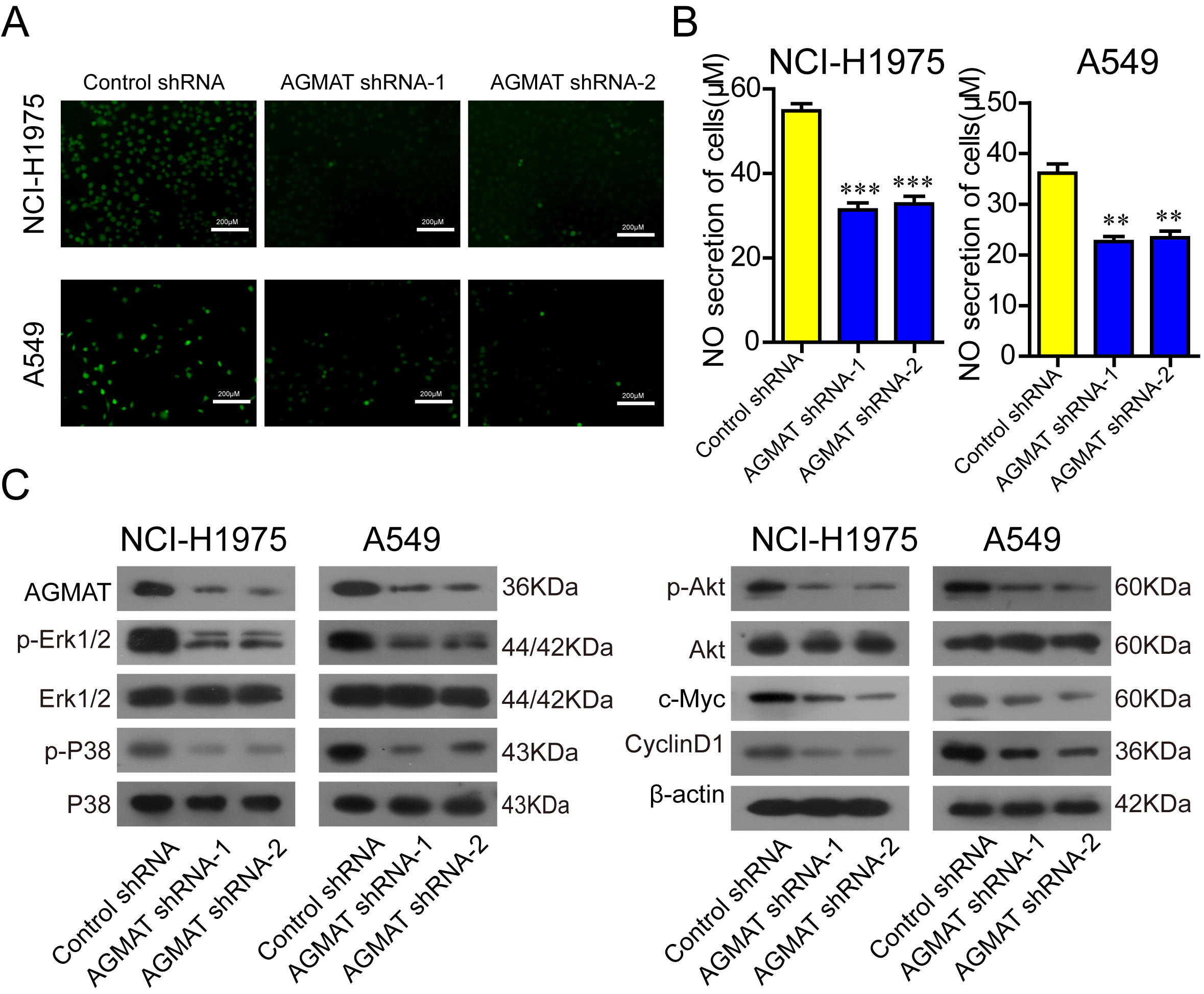

Supplement: Supplementary file 7 — Supplementary Figure 6 [file 41419_2019_2082_MOESM7_ESM.tif]
